# Application of Antithrombotic Drugs in Different Age-Group Patients with Upper Gastrointestinal Bleeding

**DOI:** 10.1155/2024/1710708

**Published:** 2024-04-04

**Authors:** Ding Peng, Huihong Zhai

**Affiliations:** Department of Gastroenterology, Xuanwu Hospital, Capital Medical University, Beijing 100053, China

## Abstract

**Objective:**

This study aimed at exploring the safety and timing of antithrombotic drugs in different age-group patients with UGIB.

**Methods:**

An observational study retrospectively based on the single-center database with 713 patients with UGIB.

**Result:**

Among the 713 patients, 62.13% were elderly patients (aged > 60 years) and the mortality was 2.9%. In elderly patients with UGIB, we found that previous medication history, resumption of medication, and time of resumption did not affect the in-hospital mortality. The resumption of anticoagulants increased the risk of rebleeding. The independent risk factors of mortality were CHF, cirrhosis, creatine kinase, and albumin. The independent risk factors of rebleeding were the application of anticoagulants during hospitalization, variceal bleeding, black stool, red blood cells (lab), platelets (lab), and heart rate.

**Conclusions:**

In UGIB patients, a history of antiplatelet or antithrombotic drugs and the use of antiplatelet drugs after UGIB did not affect the patient's prognosis. In elderly UGIB patients, although antithrombotic drugs did not increase the risk of death, the increased risk of rebleeding after resumption of use deserved careful treatment. It was safe to recover anticoagulant drugs as soon as possible in young UGIB patients.

## 1. Introduction

Antithrombotic drugs were among the most widely used drugs in the world, including aspirin, clopidogrel, and warfarin, which could reduce the occurrence of thrombotic events [1]. However, due to its side effects of increasing the risk of bleeding, there were many disputes about the application of these drugs in patients with upper gastrointestinal bleeding (UGIB), especially in the timing of the resumption of antithrombotic drugs. Considering the unique nature of medication in the elderly, it was not yet clear whether there were differences in the resumption of antithrombotic medication use among patients of different age groups. This study aimed to explore the safety and timing of antithrombotic drugs in different age-group patients with UGIB through retrospective analysis, to help clinical decision.

## 2. Materials and Methods

This is a retrospective observational study of patients based on the single-center database from a 3A hospital in Beijing, China, which was the National Clinical Research Center for Geriatric Diseases. Our study collected data from 713 UGIB patients, who was admitted to the hospital from February 2016 to January 2022. The causes of UGIB included peptic ulcer, laceration, inflammation, varicosity, and angiodysplasia. We excluded patients complicated with pregnancy, trauma, poisoning, and transplantation. The protocol for the research project has been approved by a suitably constituted ethics committee of the institution within which the work was undertaken and that it conforms to the provisions of the Declaration of Helsinki (as revised in Tokyo 2004) and that patient anonymity was preserved. The study was conducted in accordance with the Basic and Clinical Pharmacology and Toxicology policy for experimental and clinical studies [2].

We distinguished the antithrombotic drugs into antiplatelets and anticoagulants. Antiplatelet medications included ticagrelor, ticlopidine, clopidogrel, and aspirin. Anticoagulants included warfarin, heparin, and rivaroxaban. In this study, patients were divided into a young group (age 18–59) and an elderly group (age over 60).

The primary endpoint was mortality during hospitalization. The secondary endpoint was rebleeding and operative treatment. Rebleeding was defined as a decrease in hemoglobin by more than 20 g/L compared with that in admission. The operative treatment included surgical and vascular interventional procedures.

### 2.1. Statistical Analysis

R version 44.1.2 and SPSS 26.0 for Windows were used to analyze all the data. The packages of R include “tableone”, “survival”, “plyr”, “ggplot2”, and “foreign”. First, we conducted the descriptive analysis by *t*-test (normally distributed continuous variables), Kruskal test (nonnormally distributed continuous variables), or the chi-squared test (categorical variables). Univariate survival analysis was performed by plotting the Kaplan-Meier curve and log-rank test. Finally, we analyzed the independent factors by LASSO and Cox multivariate hazard regression analysis. All tests were two-sided, and a *P* value of <0.05 was considered to be statistically significant. The missing value was not interpolated.

## 3. Results

Among the 713 patients, 443 (62.13%) were elderly patients (aged > 60 years) and the mortality was 2.9% (21/713). According to Table 1 (baseline characteristics), elderly patients had the following characteristics: a high proportion of females and complications (including coronary heart disease, hypertension, diabetes, heart failure, valve disease, tumor disease, kidney disease, respiratory disease, atrial fibrillation, and cerebrovascular disease). In terms of laboratory examination, hemoglobin, platelet count, calcium ion, and albumin levels in elderly patients were low, but the levels of urea nitrogen, globulin, INR, and creatinine were high. In terms of vital signs, elderly patients had low heart rates, high blood pressure, and low BMI.

In terms of prognosis and application of antithrombotic drugs (Table 2), we found that elderly patients had a longer hospital stay, a higher proportion of patients with a history of antiplatelet drugs, and a higher proportion of patients who used anticoagulants during hospitalization. The median time for elderly patients to use antiplatelet drugs after admission was 10 [4, 14] days, and the median time for using anticoagulant drugs was 6 [3, 13] days. The median time for young patients to use antiplatelet drugs after admission was 8 [3, 13] days, and the median time for using anticoagulant drugs was 8 [4, 11] days. There was no statistical difference between the two groups in the time interval between the resumption of drug application.

Table 3 shows the influence of the application of anticoagulant/antiplatelet drugs on the prognosis of patients in the two groups. We found that the previous medication history did not affect the death, rebleeding, and length of hospital stay. In terms of medication during hospitalization, we found that elderly patients who used antiplatelet drugs had longer hospitalization time, but there was no significant difference in rebleeding rate and mortality compared to patients who did not use antiplatelet drugs. In addition to the longer hospitalization time, the rebleeding rate of the elderly patients in the anticoagulant group was also higher than that in the nonanticoagulant group. But the use of anticoagulants did not affect the death of patients. In young patients, the use of antiplatelet drugs or anticoagulants during hospitalization had no impact on the prognosis. As for the timing of the resumption of antiplatelet drugs or anticoagulant drugs, we found that whether to resume the use of drugs within 7 days did not affect the in-hospital mortality of patients.

Then, in elderly patients, we used K-M survival analysis to explore the impact of antiplatelets/anticoagulants on patient survival (Table 4) and used multivariate Cox regression analysis to find independent factors that affect the survival of elderly patients (Table 5). We found that antiplatelet/anticoagulant drugs had no effect on the survival of elderly patients with UGIB, and the independent risk factors affecting the survival of patients were CHF (HR 8.638, *P* < 0.001, 95.0% CI 3.211–8.638), cirrhosis (HR 4.443, *P* = 0.010, 95.0% CI 1.421–4.443), creatine kinase (HR 1.002, *P* < 0.001, 95.0% CI 1.001–1.002), and albumin (HR 0.916, *P* = 0.024, 95.0% CI 0.849–0.916).

In the analysis of rebleeding, we used multivariate logistic analysis to explore independent risk factors (Table 5). The independent risk factors affecting the rebleeding rate of elderly patients with UGIB were the application of anticoagulants during hospitalization (OR = 7.845, *P* = 0.004, 95% CI 1.966–7.845), variceal bleeding (OR = 33.65, *P* < 0.001, 95% CI 5.265–33.65), black stool (OR = 0.216, *P* = 0.014, 95% CI 0.064–0.216), red blood cells (laboratory examination) (OR = 1.053, *P* < 0.001, 95% CI 1.028–1.053), platelets (laboratory examination) (OR = 1.01, *P* < 0.001, 95% CI 1.004–1.010), and heart rate (OR = 0.972, *P* = 0.012, 95% CI 0.951–0.972).

## 4. Discussion

As we know, this study was the first article to report the application of antithrombotic drugs in different age-group patients with UGIB. In addition, compared with previous studies based on Asian populations, our study had a larger sample size [3]. To more comprehensively analyze the impact of anticoagulant/antiplatelet on the prognosis of patients, both the previous medication history and medication during hospitalization were considered, which previous research less reported. Finally, we found that in elderly patients with UGIB, medical history of anticoagulant/antiplatelet did not affect the prognosis of patients. The use of antiplatelet after bleeding did not increase the risk of mortality and rebleeding, while the use of anticoagulant increased the risk of rebleeding but did not increase the risk of mortality. These results would be useful in guiding clinicians to use anticoagulant/antiplatelet more safely in elderly patients with UGIB and improve the prognosis of patients.

In terms of the application of antiplatelet drugs, the latest guidelines point out that continuous use of aspirin was safe when endoscopic hemostasis was successful, but there was no conclusion on the application of P2Y12 inhibitors [4]. This article showed the safety of resuming the use of antiplatelet drugs in elderly patients with UGIB, whether aspirin or P2Y12 inhibitors. In terms of recovery time, resuming the use of antiplatelet drugs within 4–14 days was safe.

Anticoagulants were widely used to prevent thrombotic diseases, including venous thromboembolism (VTE), atrial fibrillation (AF), and mechanical heart valve prostheses [5]. However, in UGIB patients, the safety and time of the resumption of anticoagulants were still widely controversial. Observational studies generally showed higher rates of rebleeding when anticoagulants were resumed within the first 2–3 weeks after GI bleed. Conversely, the majority of thromboembolic events occurred beyond 2 weeks of hospitalization when anticoagulation was discontinued [6]. A large number of studies had shown that the resumption of anticoagulants during hospitalization significantly increased the risk of bleeding, but reduced the risk of mortality [7–9]. Based on the above research, the current guidelines in Europe, the United States, and the Asia Pacific recommend the resumption of the use of anticoagulant drugs [10–12]. However, there were still great differences in the timing of the resumption of anticoagulant drugs. In addition, there were a few studies based on the Chinese population cited in the above guidelines. Our study showed that the use of anticoagulants in elderly patients with UGIB increased the risk of rebleeding, but not in young patients. It also pointed out that it was safe to resume the use of anticoagulants in about 3–13 days after UGIB.

In exploring the independent risk factors of rebleeding and mortality, the previous study had pointed out that cirrhosis was an independent risk factor for mortality in very old patients [13]. This was consistent with the results in our article that cirrhosis or variceal bleeding was independently related to death or rebleeding. Among other factors, including black stool, heart rate, age, and heart failure, platelets were currently commonly used in gastrointestinal bleeding scores, such as GBS, RS, and AMIS65 [14–16]. It was worth noting that CK was an independent risk factor for mortality in our study, and this index was rarely mentioned to be related to gastrointestinal bleeding. As a marker of muscle injury, especially myocardial injury, CK was widely used. Research shows that UGIB was associated with a risk of cardiac injury of up to 19%. Diminished oxygen supply due to anemia and increased demand due to tachycardia were key factors for muscle injury. [17]. Myocardial injury in elderly patients often mean a worse prognosis. On the other hand, it also mean that early recovery of AP may be beneficial to reduce further thrombosis and reduce the possibility of further myocardial injury. In addition, the increase of CK may also be caused by muscle tissue hypoxia caused by anemia. In any case, further research is worth looking forward to.

In addition, in the multivariate analysis, we found that black stool and heart rate were protective factors for patients with rebleeding, while albumin was a protective factor for patient mortality. Albumin as a protective factor had been reported multiple times in previous literature, especially in UGIB patients with varicose veins [18, 19] There had been no similar reports on the correlation between black stools, heart rate, and rebleeding in UGIB patients, which may be related to the study of elderly patients in this article. We speculated that haematemesis was the most common chief complaint in UGIB patients who have not experienced black stools, and this group of people experiences faster and more urgent bleeding, with a higher rate of rebleeding.

As an observational retrospective research, we cannot entirely exclude all confounding factors or account for the absence of data. We cannot avoid selection bias caused by admission. However, we believed that the results of this study indicate the safety of antiplatelet/anticoagulant drugs in Chinese patients with UGIB. However, the use of anticoagulants in elderly patients still required vigilance against the risk of rebleeding, and its impact on the prognosis of elderly patients needs further confirmation through larger scale researches.

## 5. Conclusion

In UGIB patients, a history of antiplatelet or antithrombotic drugs and the use of antiplatelet drugs after UGIB did not affect the patient's prognosis. In elderly UGIB patients, although antithrombotic drugs did not increase the risk of death, the increased risk of rebleeding after resumption of use deserved careful treatment. It was safe to recover anticoagulant drugs as soon as possible in young UGIB patients.

## Figures and Tables

**Table 1:** Baseline characteristics.

	Young group (270)	Elderly group (443)	*P* value
Female	34 (12.6)	138 (31.2)	<0.001
Age	48.00 [39.00, 55.00]	71.00 [65.00, 80.50]	<0.001
Black stool	209 (77.4)	323 (72.9)	0.212
Haematemesis	113 (41.9)	181 (40.9)	0.855
Comorbidities			
CAD	38 (14.1)	150 (33.9)	<0.001
CHF	10 (3.7)	51 (11.5)	0.001
Hypertension	77 (28.5)	274 (61.9)	<0.001
Diabetes	44 (16.3)	176 (39.7)	<0.001
Valvular disease	11 (4.1)	76 (17.2)	<0.001
Oncological disease	13 (4.8)	95 (21.4)	<0.001
Kidney disease	12 (4.4)	60 (13.5)	<0.001
Respiratory diseases	30 (11.1)	184 (41.5)	<0.001
Cirrhosis	25 (9.3)	43 (9.7)	0.948
Atrial fibrillation	0 (0.0)	34 (7.7)	<0.001
Cerebrovascular disease	13 (4.8)	94 (21.2)	<0.001
Laboratory values			
WBC	7.36 [5.82, 9.78]	7.43 [5.58, 9.79]	0.604
Hemoglobin	86.00 [73.00, 102.00]	82.00 [70.00, 96.00]	0.018
Platelets	200.00 [163.00, 247.00]	179.00 [130.50, 222.50]	<0.001
Urea nitrogen	6.03 [4.40, 9.05]	7.80 [5.50, 12.05]	<0.001
Calcium	1.98 [1.82, 2.08]	1.93 [1.59, 2.06]	0.013
Creatine kinase	89.00 [62.50, 136.50]	80.00 [52.25, 142.00]	0.097
Globulin	20.38 [17.16, 23.21]	21.58 [17.56, 25.00]	0.007
INR	1.09 [1.05, 1.17]	1.12 [1.05, 1.18]	0.048
Creatinine	75.00 [64.00, 84.25]	78.00 [63.00, 96.00]	0.012
Albumin	34.16 [30.71, 37.29]	32.00 [28.81, 34.37]	<0.001
Physical examination			
Heart rate	88.00 [80.00, 95.00]	82.00 [76.00, 90.00]	<0.001
SBP	126.00 [115.00, 137.00]	130.00 [118.00, 141.00]	0.001
Respiratory rate	20.00 [18.00, 22.00]	20.00 [18.00, 21.00]	0.367
Height	72.15 [62.12, 84.00]	65.00 [55.00, 74.60]	<0.001
Weight	172.00 [167.00, 177.00]	168.00 [160.00, 172.00]	<0.001
BMI	24.54 [22.00, 28.34]	24.24 [21.83, 26.25]	0.022

CHF: congestive heart failure; SBP: systolic blood pressure; CAD: coronary artery disease; PVD: peripheral vascular disease; WBC: white blood cell count; INR: international normalized ratio.

**Table 2:** Prognosis and application of antithrombotic drugs.

	Young group (270)	Elderly group (443)	*P* value
Variceal hemorrhage	18 (6.7)	28 (6.3)	0.980
Interventional procedure	3 (1.1)	2 (0.5)	0.575
Endoscopy procedure	4 (1.5)	7 (1.6)	1.000
Surgical procedure	6 (2.2)	12 (2.7)	0.876
Red blood cell transfusion	70 (25.9)	182 (41.1)	<0.001
Rebleeding	5 (1.9)	22 (5.0)	0.056
Death	1 (0.4)	20 (4.5)	0.003
Resumption of antiplatelets	26 (9.6)	52 (11.7)	0.453
Resumption of anticoagulants	7 (2.6)	34 (7.7)	0.008
Medical history of antiplatelets	33 (12.2)	114 (25.7)	<0.001
Medical history of anticoagulants	1 (0.4)	11 (2.5)	0.068
Length of stay	13.00 [10.00, 16.00]	15.00 [12.00, 19.00]	<0.001

**Table 3:** Effect of antithrombotic drug application on prognosis of patients in the two groups.

		Elderly group			Young group		
		Length of stay (days)	Rebleeding	Mortality	Length of stay (days)	Rebleeding	Mortality
History of AP	Y	16.38 ± 0.57	6 (0.053%)	5 (0.044%)	14.64 ± 0.94	0 (0%)	0 (0%)
	N	15.56 ± 0.43	16 (0.049%)	15 (0.046%)	13.38 ± 0.36	5 (0.021%)	1 (0.004%)
	*P*	0.154	0.865	0.939	0.100	0.400	0.709
History of AC	Y	17.91 ± 2.06	0 (0%)	1 (0.091%)	7.00 ± 0.00	0 (0%)	0 (0%)
	N	15.72 ± 0.35	22 (0.051%)	19 (0.044%)	13.56 ± 0.34	5 (0.019%)	1 (0.004%)
	*P*	0.277	0.443	0.459	0.148	0.891	0.951
AP after UGIB	Y	18.08 ± 0.87	3 (0.058%)	2 (0.038%)	15.54 ± 1.16	0 (0%)	0 (0%)
	N	15.46 ± 0.38	19 (0.049%)	18 (0.046%)	13.32 ± 0.35	5 (0.02%)	1 (0.004%)
	*P*	0.040	0.777	0.805	0.054	0.461	0.744
AC after UGIB	Y	22.68 ± 2.27	6 (0.176%)	3 (0.088%)	13.86 ± 2.11	0 (0%)	0 (0%)
	N	15.2 ± 0.31	16 (0.039%)	17 (0.042%)	13.52 ± 0.35	5 (0.019%)	1 (0.004%)
	*P*	<0.001	<0.001	0.208	0.609	0.713	0.870

**Table 4:** Log-rank test of antithrombotic drugs on elderly patient death.

	Log-rank	*P* value
History of AP	0.009	0.926
History of AC	0.475	0.291
AP after UGIB(Y vs. N)	0.423	0.515
AC after UGIB(Y vs. N)	1.563	0.211
AP after UGIB(0–7 days vs. >14 days)	0.005	0.941
AC after UGIB(0–7 days vs. >14 days)	0.032	0.857

**Table 5:** Multivariate hazard regression analysis.

	*B*	*P* value	OR	95.0% CI–OR
Logistic analysis of rebleeding				
Resumption of anticoagulants	2.060	0.004	7.845	1.966–7.845
Variceal hemorrhage	3.516	<0.001	33.65	5.265–33.650
Black stool	−1.533	0.014	0.216	0.064–0.216
Hemoglobin (laboratory examination)	0.052	<0.001	1.053	1.028–1.053
Platelets (laboratory examination)	0.01	<0.001	1.010	1.004–1.010
Heart rate	−0.028	0.012	0.972	0.951–0.972
Cox analysis of death				
CHF	2.156	<0.001	8.638	3.211–8.638
Cirrhosis	1.491	0.010	4.443	1.421–4.443
Creatine kinase	0.002	<0.001	1.002	1.001–1.002
Albumin	−0.088	0.024	0.916	0.849–0.916

CHF: congestive heart failure.

## Data Availability

The data of this study are available from the corresponding author upon reasonable request.
